# Mechanical Stimulation and Aligned Poly(ε-caprolactone)–Gelatin Electrospun Scaffolds Promote Skeletal Muscle Regeneration

**DOI:** 10.1021/acsabm.4c00559

**Published:** 2024-10-04

**Authors:** Francisco José Calero-Castro, Víctor Manuel Perez-Puyana, Imán Laga, Javier Padillo Ruiz, Alberto Romero, Fernando de la Portilla de Juan

**Affiliations:** † Department of General and Digestive Surgery, “Virgen del Rocío” University Hospital/IBiS/CSIC/University of Seville, 41013 Seville, Spain; ‡ Oncology Surgery, Cell Therapy, and Organ Transplantation Group. Institute of Biomedicine of Seville (IBiS), “Virgen del Rocío” University Hospital, IBiS, CSIC/University of Seville, 41013 Sevilla, Spain; § Departamento de Ingeniería Química, Facultad de Química, Universidad de Sevilla, 41012 Sevilla, Spain

**Keywords:** scaffolds, biomaterials, electrospinning, PCL, gelatin, skeletal muscle

## Abstract

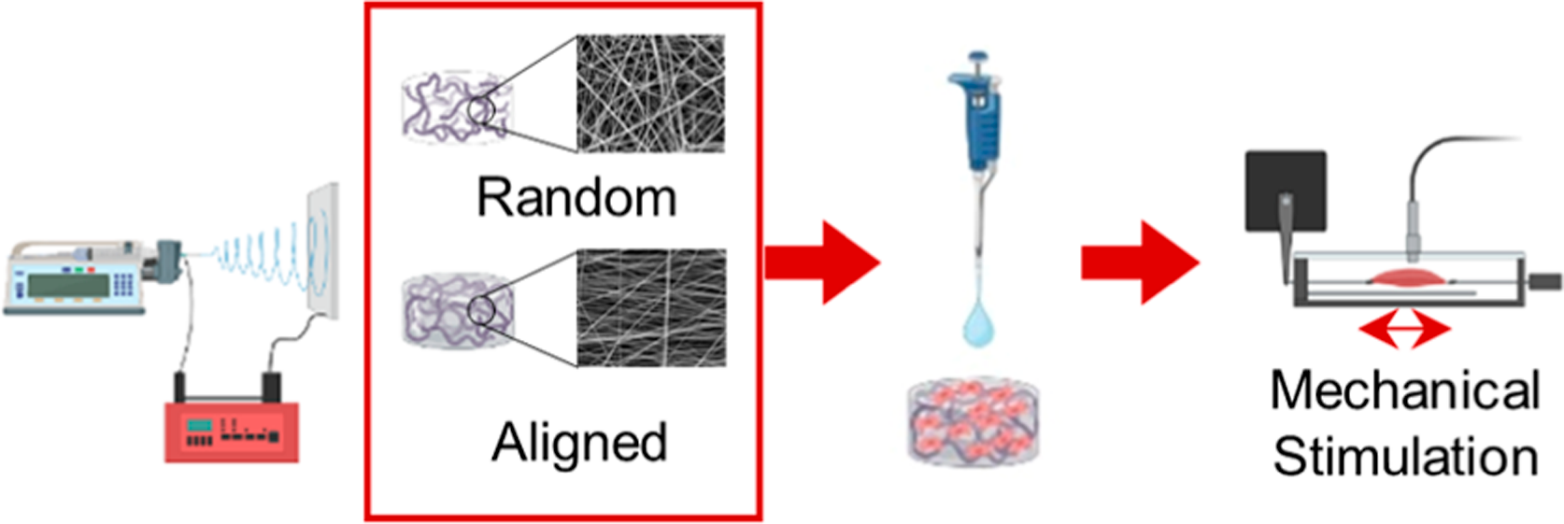

The current treatments to restore skeletal muscle defects present several injuries. The creation of scaffolds and implant that allow the regeneration of this tissue is a solution that is reaching the researchers’ interest. To achieve this, electrospinning is a useful technique to manufacture scaffolds with nanofibers with different orientation. In this work, polycaprolactone and gelatin solutions were tested to fabricate electrospun scaffolds with two degrees of alignment between their fibers: random and aligned. These scaffolds can be seeded with myoblast C2C12 and then stimulated with a mechanical bioreactor that mimics the physiological conditions of the tissue. Cell viability as well as cytoskeletal morphology and functionality was measured. Myotubes in aligned scaffolds (9.84 ± 1.15 μm) were thinner than in random scaffolds (11.55 ± 3.39 μm; *P* = 0.001). Mechanical stimulation increased the width of myotubes (12.92 ± 3.29 μm; *P* < 0.001), nuclear fusion (95.73 ± 1.05%; *P* = 0.004), and actin density (80.13 ± 13.52%; *P* = 0.017) in aligned scaffolds regarding the control. Moreover, both scaffolds showed high myotube contractility, which was increased in mechanically stimulated aligned scaffolds. These scaffolds were also electrostimulated at different frequencies and they showed promising results. In general, mechanically stimulated aligned scaffolds allow the regeneration of skeletal muscle, increasing viability, fiber thickness, alignment, nuclear fusion, nuclear differentiation, and functionality.

## Introduction

Skeletal muscle represents about 40% to 50% of the total weight of the human being and contributes significantly to several bodily functions. Its main function is the transformation of chemical energy into mechanical energy.^1^ This tissue can self-repair and regenerate in response to any injury or damage. However, when muscle loss exceeds 20% and the lesion is several, also known as volumetric muscle loss (VML), regeneration is not possible.^2,3^ Currently, the conventional strategy of care for VML is to use autologous muscle grafts. However, this treatment has different limitations, such as tissue availability, the grafted muscle flap does not restore lost functionality, postoperative infection, scar tissue formation, and donor site morbidity.^4^ This is the reason why researchers have increased their interest in the field of tissue regeneration.

Tissue engineering (TE) emerges as a promising approach for the development of novel strategies to repair VML by mimicking the properties of native tissue.^5^ This field uses stem cells, biomaterial scaffolds, and bioactive molecules to engineer functional tissue. The use of scaffold-associating cells mimics the tissue environment and the factors that stimulate the cells in their proliferation and differentiation.^6^

One of the most promising techniques for the creation of scaffolds is electrospinning. This approach lets us get scaffolds with a fibrillar conformation that improves the regeneration of different tissues^7^ such as tendon regeneration,^8^ cartilage repair^9^ or cardiac TE,^9^ and different medical applications. This technique has attracted the interest of many researchers due to its ability to create 3D porous nanofibrous scaffolds with properties similar to those of human tissue and the native extracellular matrix (ECM) network at the nanoscale level.^10^ Electrospun structures present a very high surface area, making these structures a suitable candidate for cell adhesion, proliferation, and differentiation, which is essential to guide tissue formation.^11^ Several parameters can be adjusted during electrospinning. Fiber thickness and fiber morphology can be modified by varying different electrospinning parameters such as the type of polymer, the nature of the solvent, the applied voltage, and the distance between the syringe tip and the type of collector.^10^ For example, a rotating type collector allows obtaining aligned nanofibers, while the static type collector favors the generation of random nanofibers.^7^ It has been demonstrated that the rotational speed of the collector had a considerable impact on the anisotropy of the resulting fiber mesh, which in turn influenced the mechanical properties of the scaffolds and the orientation and rearrangement of the nanofibers.^12^ Choi et al.^13^ compared the influence of unidirectional fiber orientation with nonoriented fibers. They showed that scaffolds with a unidirectional fiber orientation play a role in cell orientation and enhance myotube formation. Different works demonstrated that the alignment of fibers in these scaffolds favors cytoskeleton reorganization in myoblasts in skeletal muscle regeneration.^13−16^

Poly(ε-caprolactone) (PCL) is one of the most widely used biodegradable polymers in electrospinning and was approved by the FDA for use in various biomedical applications ranging from drug delivery to implantable devices.^17^ However, PCL has some drawbacks, such as its hydrophobicity and low degradation rate, which limit its cellular interaction and postimplantation resorption, respectively. Therefore, it has been used in combination with other materials to improve its biological and mechanical properties.^18−21^ One of these materials that has been combined with PCL is gelatin, which is a denatured, hydrolyzed form of collagen obtained by hydrolyzing collagen protein fibrils through physical and chemical methods. Gelatin has been used in various forms and blends for the manufacture of scaffolds^22^ for TE applications due to its biodegradability, biocompatibility, and simple processing properties.^23^ Finally, there are different studies that demonstrate that the combination of 4% gelatin and 16% PCL is ideal for use in the creation of scaffolds since it creates more deformable, less rigid, and more hydrophilic structures, favoring greater cell adhesion.^24,25^

Another important element of TE is the use of bioreactors with stimulation to recreate the physiology of the tissue that can distribute the cells homogeneously and increase cell proliferation, maturation, differentiation, and functionalization.^26^ Many works have described improved tissue functionality and differentiation when scaffolds are exposed to mechanical stimulation,^27,28^ increasing actin and myosin expression,^29,30^ as well as diameter and length of mature skeletal muscle fibers.^31^ Several studies have shown a pattern of mechanical stimulation in which crops were subjected to a stress load that increased over time. In addition, these loads were accompanied by rest.^32−34^ Studies using mechanical stimulation for tissue-engineered skeletal muscle structures have demonstrated improvements in differentiation, maturation, alignment, and contractility of tissue-engineered muscle.^3^

Our previous work demonstrated good behavior of PCL and gelatin scaffolds,^24^ supporting this project that aims to combine these scaffolds with mechanical stimulation in a bioreactor. Here, we hypothesize that electrospun scaffolds of PCL with gelatin, along with their stimulation in a mechanical bioreactor, could improve the creation of skeletal muscle tissue. This project aims to regenerate skeletal muscle using PCL/gelatin electrospun scaffolds and mechanical stimulation. To demonstrate this, we measured the viability, morphology, cytoskeleton, and functionality of the myotubes.

## Materials and Methods

### Fabrication of Scaffolds

**Figure 1:**
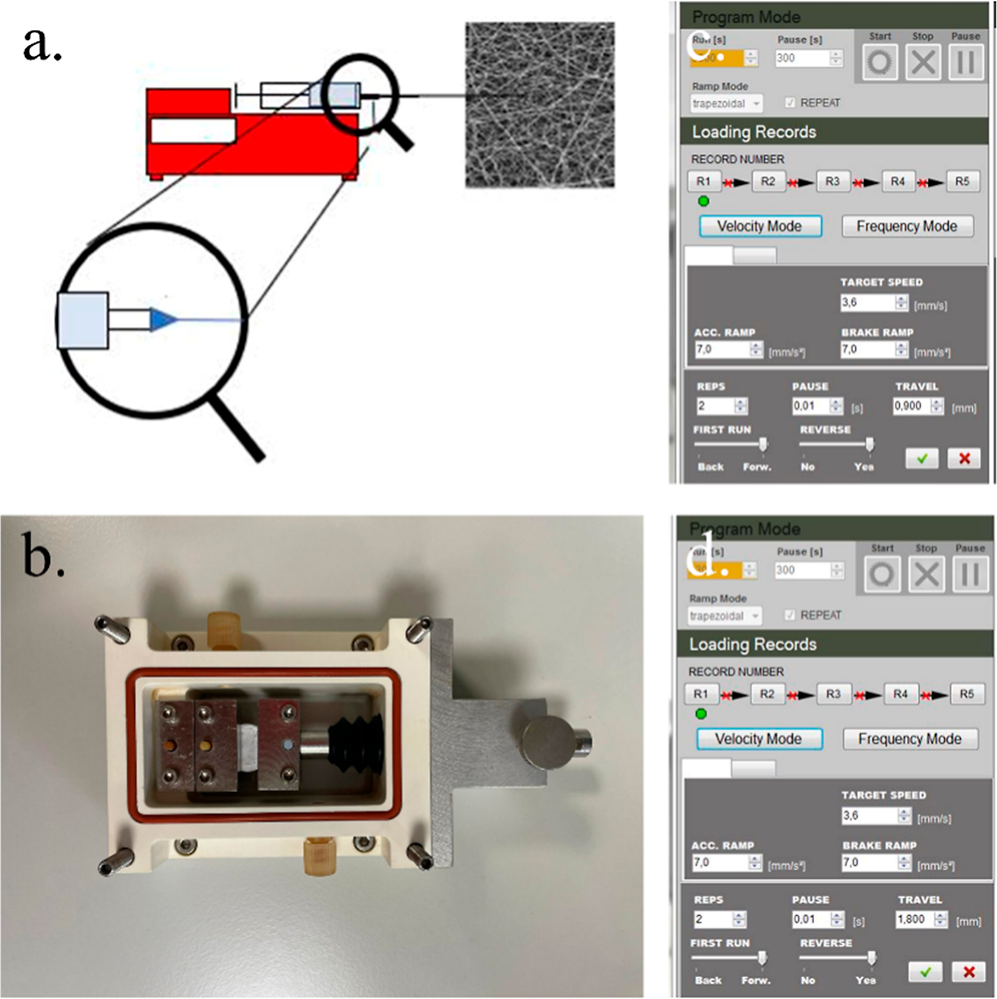
(a) Schematic image of the electrospinning setup. (b) Mechanical bioreactor with electrospun scaffolds. (c,d) Software setup for mechanical stimulation.

Electrospun scaffolds were fabricated using a binary solution based on PCL (Sigma-Aldrich, Germany) in 16% yield and type B gelatin protein (80–120 gBloom, Henan Boom Gelatin Co., Ltd., China) in a 4% yield. The electrospinning process was carried out with electrospinning equipment Fluidnatek LE-50 (Bioinicia, Valencia, Spain) (Figure 1). The process was conducted in vertical mode with the following conditions: 14 kV, 0.4 mL h-1, 14 cm gap, and 40% environmental humidity (an 18G stainless steel needle was used). Two different scaffolds were processed by changing the rotational speed of the collector, obtaining scaffolds with a random fiber orientation (with no rotational speed) and scaffolds with an aligned fiber orientation (with a rotational speed higher than 500 rpm). The scaffolds fabricated presented a circular shape with an 18 mm diameter and 10–15 μm thickness.

### Characterization of Scaffolds

#### SEM Imaging

The microscopic examination of the scaffolds was performed using a Zeiss EVO scanning electron microscope (Germany) with a secondary electron detector at an acceleration voltage of 10 kV. SEM images were obtained at two different magnifications (1000× and 4000×). A digital processing free software (ImageJ) was used to determine the mean fiber size of the nanostructures.

#### Contact Angle Measurements

Scaffold wettability was obtained by water contact angle measurements using a Drop Shape Analyzer (Germany). It was calculated as the mean value of the right and left sides of a 5 μL deionized water drop after 5 s of stability after droplet deposition.

#### Mechanical Properties

The static tensile test of the nanofibrous membranes was carried out on a RSA3 rheometer (TA Instruments, USA). Tensile tests were carried out at 1 mm/min on samples with a rectangular shape. From each test, the different parameters, including the maximum stress, strain at break, and Young’s modulus, were calculated.

### Cell Culture

The C2C12 skeletal muscle cell line (ATCC CRL-1458) was used for the cell culture. The cells were cultured in a growth medium (GM) composed of Dulbecco’s modified Eagle medium (DMEM)/high glucose (SH30285.01, HyClone) supplemented with 10% FBS (F7524, Sigma-Aldrich) and 1% penicillin–streptomycin (15140-122, Gibco) at 37 °C in an atmosphere of 5% CO_2_ until they reached a confluence of 70–80%. The cells were split by using 0.05% trypsin/EDTA (25300-062, Gibco) for subsequent seeding on the scaffolds. The myoblasts were seeded on the scaffolds in a cell concentration of 10 × 10^6^ cells per scaffold based on previous results of our group.^25^ The scaffolds were cultured on a 6-well plate with the GM at 37 °C in an atmosphere of 5% CO_2_ and they were cultured for 2 weeks. After 4 days of seeding, the GM was replaced by the differentiation medium (DM) DMEM/high glucose supplemented with 2% FBS and 1% penicillin–streptomycin. The medium was regularly replaced every 48 h.

### Mechanical Stimulation in a Bioreactor

Random and aligned scaffolds were cultured with mechanical tensile stimulation after 48 h from scaffold seeding with the TC3 bioreactor (EBERS Medical Technology SL, Zaragoza, Spain) for 2 weeks in an incubator at 37 °C in an atmosphere of 5% CO_2_ (Figure 1b) in the same conditions, 4 days with the GM and with the DM up to 2 weeks after seeding. Briefly, the scaffolds are placed between two grips in order to fix the tissue sample to the chambers; one of them was fixed and the other one was moving, applying the stimulation to the scaffolds. The stimulation was controlled by the EBERS software of the bioreactor (Figure 1c,d). The amplitude varied over the 2 weeks of culture: 5% amplitude of the scaffold long (0.9 mm) during week 1, followed by mechanical stimulation with 10% amplitude (1.8 mm) during week 2. The scaffolds were stimulated for 55 and 5 min of rest in relaxation at a frequency of 0.5 Hz for 2 weeks based on previous works.^3^

### Viability

The cell viability was measured on day 14 using a live/dead cell viability kit (consisting of calcein/ethidium (EthD-1); LIVE/DEAD Viability/Cytotoxicity Kit, Invitrogen). The medium was removed, and the scaffolds were washed with PBS. We diluted EthD-1 2 mM and calcein AM 4 mM solution in PBS to get a dilution of 4 and 2 μM, respectively, and shook to homogenize the reagent. We stained each scaffold with 200 μL of the prepared solution and incubated it for 30 min. We visualized the cells using a Nikon A1R^+^ confocal microscope with a 488 nm laser at a 10× magnification and at least 5 images per scaffold were taken. Live cells were counted as green cytoplasmic, while dead cells were counted as red-stained nuclei using ImageJ. To count confluent cells, we used the watershed algorithm. The viability was defined as the ratio between the number of viable cells and total cell number (viable cells and dead cells).

### Immunofluorescence

After 2 weeks of seeding, the scaffolds were washed with PBS at least three times. We fixed the scaffolds for 15 min with paraformaldehyde and then the scaffolds were washed with PBS three times again. Subsequently, the cells were permeabilized with 0.5% Triton in PBS for 5 min, the supernatant was removed, and a blocking solution (BS; 1% bovine serum albumin, 0.1% Tween 20 in PBS) was applied for 10 min. The scaffolds were stained with Phalloidin-iFluor 647 Reagent (ab176759, Abcam, Cambridge, UK) at a concentration of 1:1000 in the BS for 1 h in a humid dark chamber with shaking at room temperature. After that, we removed and washed with BS for 2 min three times. We mounted the scaffolds on slides and added a mounting medium with DAPI (ProLong, Thermo Fisher, Waltham, Massachusetts, USA). The stained nuclei and actin of the formed cytoskeleton were visualized using a Nikon A1R + confocal microscope with 405 and 638 nm, respectively, and at least 5 images were taken per scaffold at 10× magnification and at 40× magnification. The images were analyzed with ImageJ. To measure the cell alignment, images with 10× magnification and the orientation plugin were used. Myotube orientation was calculated as % of myotubes with an orientation between −10° and 10° over the total number of myotubes in the scaffold. The fiber thickness was measured using the straight command, each fiber was measured in 5 different points, and at least 20 fibers were measured per scaffold. The fusion rate was defined as the number of nuclei possessed by the fibers over the total nuclei, and the 40× magnification images were used. The nucleus morphology (measured by circularity and aspect ratio in ImageJ) was measured using 40× magnification images. The formula for circularity is 4 x π x area/perimeter^2^ and the aspect ratio was defined as the ratio between the length of the longest line and the length of the shortest line across the nuclei.

### Functionality Assay

Cells were stained with the Fluo-4 AM kit (Invitrogen TM, Waltham, Massachusetts, USA) to measure the functionality and contractile capacity of the cultured scaffolds. The reagent was used in a 1–5 μM concentration diluted in a culture medium. The scaffolds were incubated with the solution for 20 min until the assay. Two buffer solutions were required, one as a control, characterized by a low potassium solution, and another with high potassium levels that stimulate cell contraction. Both solutions were composed of calcium chloride (C1016, Sigma-Aldrich, St. Louis, USA) at 2.5 mM, magnesium chloride (M8266, Sigma-Aldrich, St. Louis, USA) at 2 mM, HEPES (A3724, Panreac Química, Spain) at 10 mM, and glucose (G8270, Sigma-Aldrich, St. Louis, USA) at 10 mM. Both differed in sodium chloride (S9888, Sigma-Aldrich, St. Louis, USA) at 140 and 30 mM, in the control and high potassium solution, respectively, and in potassium chloride (P3911, Sigma-Aldrich, St. Louis, USA) at 2.5 and 110 mM, in the control and high potassium solution, respectively. The solutions were adjusted to pH 7.4. During the study, two syringes were used, one with each solution. Each syringe has a stopcock; the one with the control solution was always open during the whole experiment, while the one with the high potassium solution was opened at specific times to evaluate the cellular response to the stimulus. Each scaffold was stimulated at least twice. The level of luminescence varies as calcium enters the cells due to the influence of the high potassium solution; calcium binds to Fluo-4 and causes luminescence peaks. The high potassium solution can prevent the outflow of potassium, which depolarizes the cell and forces the opening of calcium channels. The 10× magnification of the Nikon A1R^+^ confocal microscope with a 488 nm laser was used for this study. Finally, we measure the luminescence cell as a function of time. The luminescence increase, which was defined as the difference in luminescence between the peak of the stimulus and the mean luminescence of the cell at rest, was measured, and the luminescence difference was defined as the difference between the first and second peaks of the stimulus. Both the luminescence increase and the luminescence difference between the peaks of each stimulus are dimensionless variables.

### Electrical Stimulation

The scaffolds that presented a high response to high potassium solution were electrically stimulated with the TC3 bioreactor signal generator (EBERS Medical Technology SL, Zaragoza, Spain) at different frequencies and visualized under confocal microscopy with Fluo-4 staining. The frequencies picked up were 0.2, 0.5, 1, 1.5, and 2 Hz.

### Statistics

Quantitative variables were summarized by the mean and standard deviation. Sample normality was also calculated by the Shapiro–Wilk test if *n* < 50 or Kolmogorov–Smirnov test if *n* > 50. For quantitative variables, the Mann–Whitney *U* test was used when the samples did not show a normal distribution. For quantitative variables that showed a normal distribution, the Student’s *t*-test was used. For the comparison of more than two samples with a normal distribution, the analysis of variance (ANOVA) test was used for independent measures, and the Kruskal–Wallis test when there was no normal distribution. The ANOVA test was used for quantitative variables when the independent variable offered more than two values, using Tukey’s test if differences were found and the variances were equal. In the case where the variances were different, Tamhane’s test was used. For the pairwise analysis of the samples that did not have a normal distribution, the Mann–Whitney *U* test was used. A *P* < 0.05 was established as statistically significant; significant: 0.01 < *P* < 0.05; very significant: 0.001 < *P* < 0.01, and extremely significant: *P* < 0.001. The IBM SPSS Statistics 19 package was used for statistical analysis. GraphPad Prism 9 and OriginPro 2022 software were used for the graphs.

## Results

### Characterization of the Scaffolds

**Figure 2:**
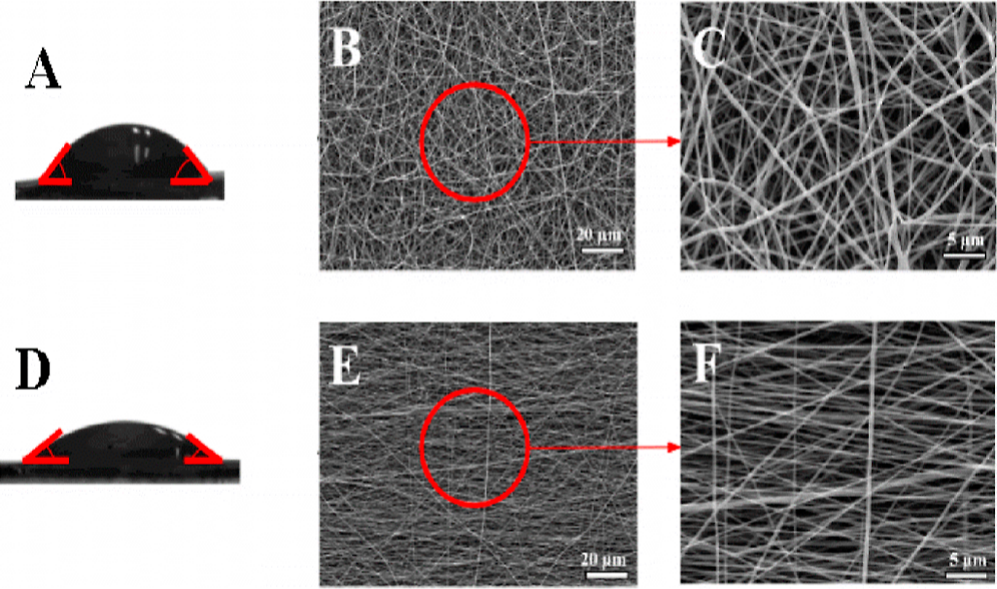
Water contact angle of the nanofiber scaffolds with (A) random and (D) aligned orientation and SEM imaging at different magnifications (1000× and 4000×) of the nanofiber scaffold with a random (B,C, respectively) and aligned orientation (E,F, respectively).

SEM images of the obtained scaffolds at different magnifications are shown in Figure 1. The different orientations obtained can be seen for both systems, from the randomly oriented system (Figure 2b,c) with fibers without a predefined orientation and from the aligned system (Figure 2d,e) with a predefined orientation toward the *X* axis. A summary of the properties of the obtained scaffolds is shown in Table 1. Water contact angle values obtained for both systems revealed a hydrophilic system with a higher wettability for the aligned system, as shown by a decrease in the contact angle (Figure 2a,b). Furthermore, the alignment of the fibers produced a system with a lower Young’s modulus and a lower deformability. On the other hand, comparing the mean fiber size (Table 1), higher fiber sizes were produced with a random orientation, although there are no significant differences between both systems.

**Table 1:** Contact Angle, Mean Fiber Size (Width), Young’s Modulus, and Strain at Break of the Different Scaffolds

	random orientation	aligned orientation
contact angle (°)	51 ± 1	31 ± 2
mean fiber size (nm)	320 ± 79	205 ± 41
Young’s modulus (Pa)	6.3·106 ± 1.5·106	2.3·106 ± 0.4·106
strain at break (mm/mm)	0.66 ± 0.05	0.31 ± 0.02

### Viability

**Figure 3:**
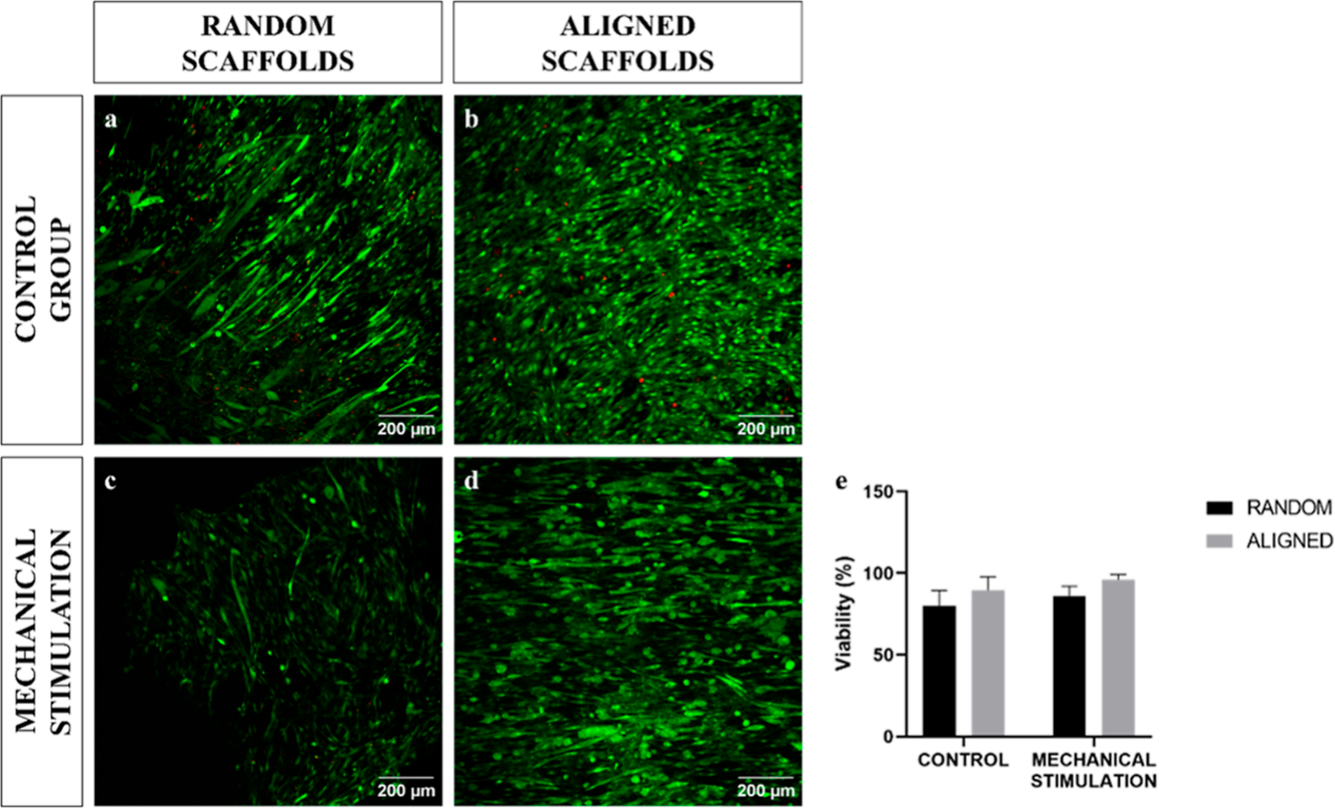
Viability of electrospun scaffolds at day 14: control random scaffolds (a), control aligned scaffolds (b), mechanically stimulated random scaffolds (c), mechanically stimulated aligned scaffolds (d), and viability (e) (scale bar: 200 μm).

The random scaffolds (*n* = 3) had a viability of 86.54 ± 9.38% (Figure 3a), while that of the aligned scaffolds (*n* = 3) was 89.47 ± 8.35% (Figure 3b). When the scaffolds were mechanically stimulated, the random scaffolds (*n* = 3) had a viability of 86.19 ± 5.93% (Figure 3c), while aligned scaffolds (*n* = 3) increased the viability to 96.00 ± 3.13% (Figure 3d). No significant differences were found between random and aligned scaffolds without stimulation (*P* = 0.267) and between random and aligned scaffolds with mechanical stimulation (*P* = 0.064). Mechanical stimulation did not lead to significant differences in cell viability in random scaffolds (*P* = 0.398) or aligned scaffolds (*P* = 0.273) (Figure 3e).

### Morphological Characterization

**Figure 4:**
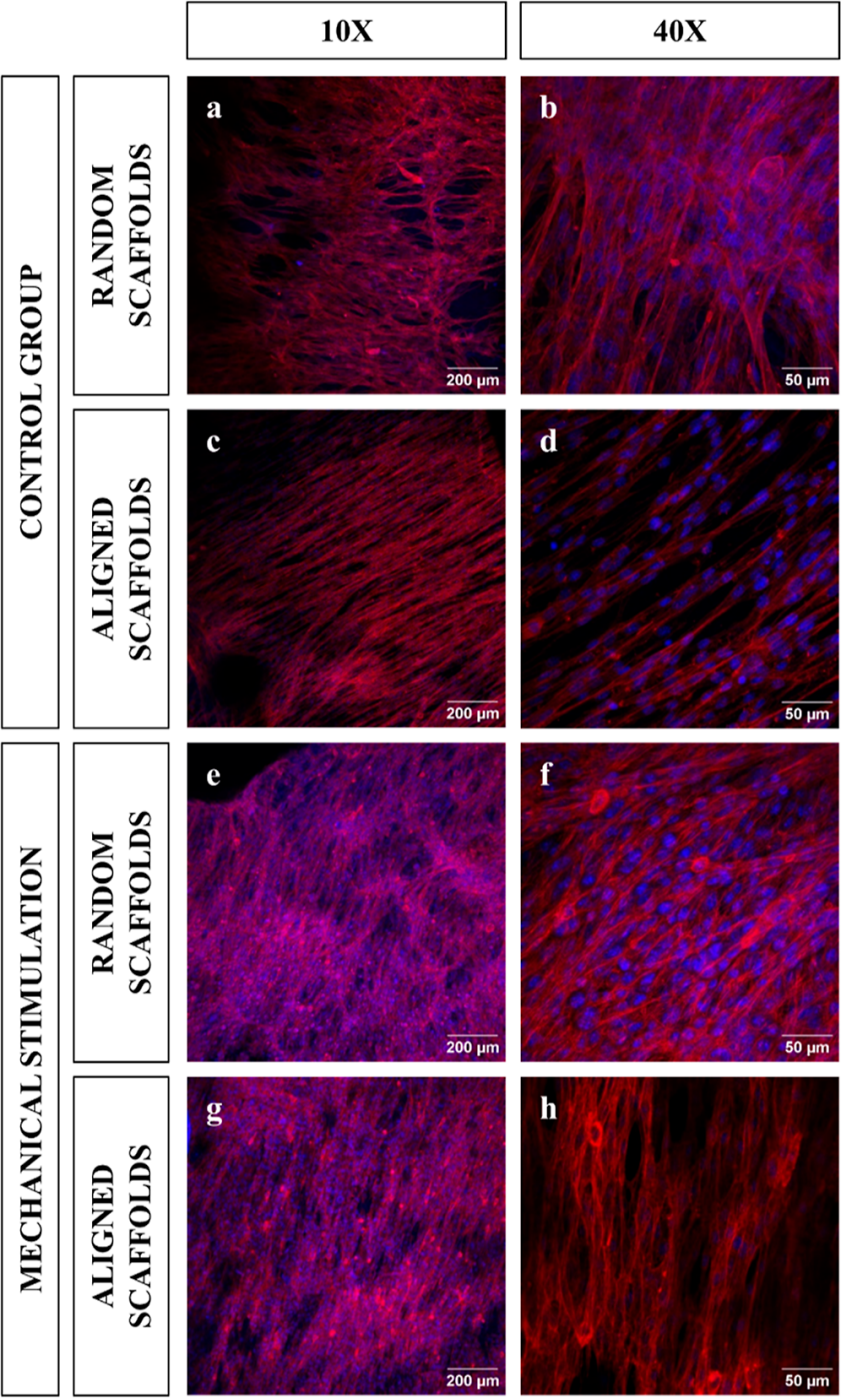
Immunostaining of myotubes after day 14. Blue: nucleus. Red: actin. Control random scaffolds 10X (a), control random scaffolds 40X (b), control aligned scaffolds 10X (c), control aligned scaffolds 40X (d), mechanical stimulated random scaffolds 10X (e), mechanical stimulated random scaffolds 40X (f), mechanical stimulated aligned scaffolds 10X (g), and mechanical stimulated aligned scaffolds 40X (h).

**Figure 5:**
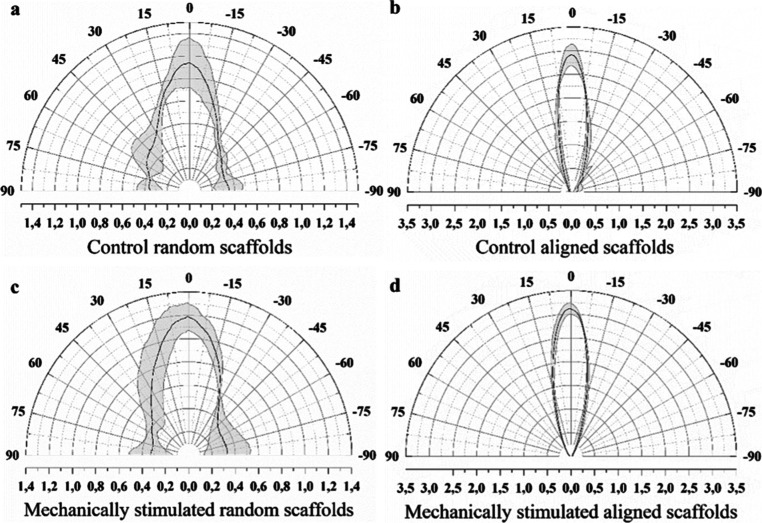
Myotube alignment at day 14. Control random scaffolds (a), control aligned scaffolds (b), mechanical stimulated random scaffolds (c), and mechanical stimulated aligned scaffolds (d).

**Figure 6:**
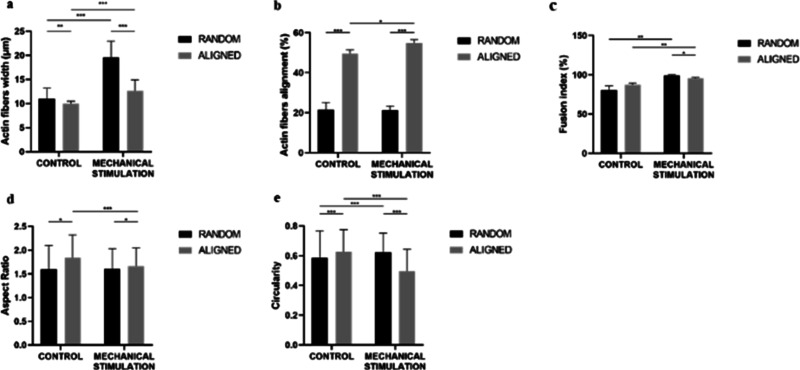
Analysis of myofibers at day 14. Actin fiber alignment (a), actin fiber width (b), fusion index (c), aspect ratio (d), and circularity (e).

Figure 4 shows the immunostaining of myotubes created with the scaffolds. Figure 5 shows the distribution of the myotubes according to each type of scaffold and stimulation used (*n* = 3). In this way, we see how the fiber orientation undergoes greater ordering as the scaffold nanofibers increase their orientation. In addition, mechanical stimulation increases the fiber ordering in the aligned scaffolds. The fiber alignment of the random scaffolds (Figure 5a) was 21.44 ± 3.71%, while the aligned scaffolds presented an alignment of 49.51 ± 2.03% (Figure 5b). The mechanically stimulated random scaffolds (*n* = 3) showed a fiber alignment of 21.07 ± 4.76% (Figure 5c), whereas the fibers of the mechanically stimulated aligned scaffolds (*n* = 3) showed an alignment of 54.89 ± 1.63% (Figure 5d). Statistical difference (Figure 6a) was found between the alignment of the myotubes of the random and aligned scaffolds without stimulation (*P* < 0.001). A significant difference (Figure 6a) was demonstrated in the alignment of myotubes of random and mechanically stimulated aligned scaffolds (*P* < 0.001). Mechanical stimulation did not induce an increase in alignment within the random scaffolds (*P* = 0.700). However, in the aligned scaffolds, stimulation was shown to promote an increase in fiber alignment (*P* = 0.023).

The thickness of the myotubes (Figure 6b) created with the random scaffolds (*n* = 3) was 11.55 ± 3.39 μm and that of the myotubes of the aligned scaffolds (*n* = 3) was 9.84 ± 1.15 μm. The existence of a significant difference between both groups was demonstrated (*P* = 0.006). The mechanically stimulated random scaffolds (*n* = 3) generated myotubes with a thickness of 19.21 ± 4.88 μm and the aligned scaffolds (*n* = 3) with a thickness of 12.92 ± 3.29 μm. A statistically significant difference between both groups (*P* < 0.001) was evidenced. In addition, it was demonstrated that mechanical stimulation increased the thickness of the myotubes in both random (*P* < 0.001) and aligned scaffolds (*P* < 0.001).

We analyzed the nucleus that were inside the fibers out of the total nucleus detected in each type of scaffold (*n* = 3). Thus, we found that in the random scaffolds, 80.73 ± 5.21% of the nucleus were inside the fibers, while in the aligned scaffolds, the density increased to 87.28 ± 2.22%. No significant difference was evident between the nuclear density of the 2 types of scaffolds (*P* = 0.116) (Figure 6c). As for the mechanically stimulated scaffolds, the random scaffolds presented a melting rate of 99.23 ± 0.89% and that of the aligned scaffolds was 95.73 ± 1.05%. There was a significant difference between both groups (*P* = 0.012). In addition, mechanical stimulation was found to increase the fusion index of both random (*P* = 0.004) and aligned scaffolds (*P* = 0.004).

To measure cell maturation, we used 2 parameters: nucleus aspect ratio (Figure 6d) and nucleus circularity (Figure 6e). Starting with random scaffolds (*n* = 3), these had a nucleus aspect ratio of 1.49 [1.30, 1.75] and a circularity of 0.59 [0.46, 0.74], while the aspect ratio was 1.76 [1.50, 2.10] in aligned scaffolds (*n* = 3) and circularity 0.65 [0.52, 0.75]. Significant differences were found between both groups in aspect ratio (*P* < 0.001) and circularity (*P* < 0.001). The myotube nucleus of the mechanically stimulated random scaffolds had an aspect ratio of 1.51 [1.29, 1.81] and a circularity of 0.62 [0.53, 0.72] and those of the aligned scaffolds had an aspect ratio of 1.60 [1.40, 1.88] and a circularity of 0.50 [0.39, 0.60]. Significant differences were evident between the aspect ratio (*P* < 0.001) and circularity (*P* < 0.001) of both scaffolds when stimulated. Finally, random scaffolds only showed a significant difference in circularity (*P* < 0.001) between the control group and the mechanically stimulated group. The aligned scaffolds presented significant differences in aspect ratio (*P* < 0.001) and circularity (*P* < 0.001).

### Functionality Assay

**Figure 7:**
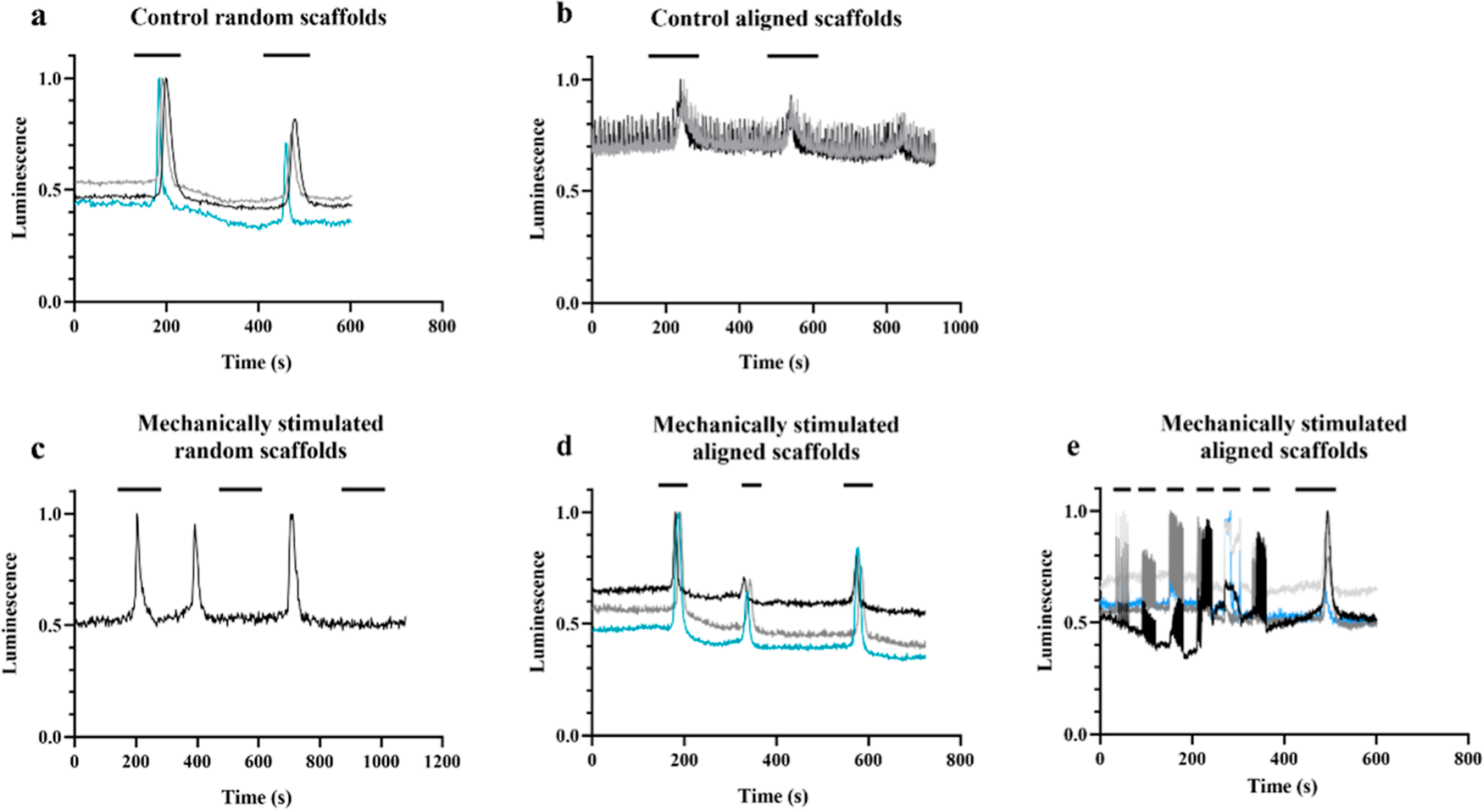
Functionality assay measured by cellular luminescence due to calcium transients at day 14 after stimulation with high potassium solution. Control random scaffolds (a), control aligned scaffolds (b), mechanically stimulated random scaffolds (c), mechanically stimulated aligned scaffolds (d), and electrical stimulation of mechanical stimulated aligned scaffolds (e).

The scaffolds (*n* = 3) were analyzed with a functionality assay. All groups showed cell functionality, although not all of it was due to the response to K^+^ ions or electrical stimulation, as shown in Figure 7, where luminescence normalized is represented. In random (Figure 7a), aligned (Figure 7b), and aligned mechanically stimulated (Figure 7d) scaffolds, some luminescence peaks can be observed approximately 1’5 min after the stimulus with high potassium solution.

In random scaffolds, the luminescence levels increased after the first stimulus of 34.75 ± 15.87 and the second stimulus of 15.01 ± 11.92. Therefore, the difference in luminescence levels is 19.74 ± 15.49. On the other hand, aligned scaffolds showed a luminescence increase of 22.37 ± 8.51 and 13.76 ± 9.26 after the first and second stimulus, respectively. The difference in luminescence between both peaks was 8.61 ± 1.54. Random and aligned scaffolds only showed significant differences related to the difference in luminescence between both peaks (*P* = 0.004), the luminescence after the first stimulus (*P* = 0. 083), and the luminescence after the second stimulus (*P* = 1.000).

The mechanically stimulated random scaffolds showed an increase in the luminescence of 25.89 ± 14.50. After the second stimulus, the increase in the luminescence was 15.37 ± 12.76. This low response may be due to the short exposure time or cell fatigue after the first stimulus. The difference in luminescence between both peaks was 10.53 ± 15.57. The mechanically stimulated aligned scaffolds showed an increase in luminescence of 30.41 ± 13.79. After the second stimulus, the increase in luminescence was 0.33 ± 3.23. This low response may be due to the short exposure time or the cell fatigue after the first stimulus. The difference in luminescence between both peaks was 30.73 ± 13.22. In mechanically stimulated scaffolds, there were significant differences in the increase in luminescence levels after the first (*P* = 0.010) and second (*P* = 0.001) stimulus, as well as in the difference between both of them (*P* = 0.001).

Mechanical stimulation in random scaffolds did not show a significant difference in the luminescence of the first stimulus (*P* = 0.136) as well as the second stimulus (*P* = 0.881), in the difference between both increases (*P* = 0.137).

Mechanical stimulation in aligned scaffolds showed a significant difference in the difference between both increases (*P* < 0.001). However, the luminescence of the first stimulus (*P* = 0.661) as well as the second stimulus (*P* = 0.054) did not show a significant difference.

Finally, since mechanically stimulated scaffolds showed evident contractility and fiber orientation, they were electrically stimulated to evaluate their response against this new stimulus (S1). As shown in Figure 7e, the applied frequencies were 0.2, 0.5, 1, 1.5, and 2 Hz for 30 s each. Cells showed a good response at lower frequencies, while at 2 Hz, their response was not as evident. This may be because the membrane depolarization may be slower than the changing rate of the applied stimuli, and the calcium ions do not enter the cells. However, after another stimulation of 1 Hz and with high potassium solution, cell functionality is again observed.

## Discussion

Nanofiber scaffolds have been extensively studied in TE. These scaffolds could recreate tissue architecture and ECM and further serve as regulators of cellular responses. It is well-known that synthetic materials are biocompatible, but upon degradation, the products generated induce an inflammatory response due to the pH change, whereas natural materials have superior biocompatible properties but weaker mechanical properties. Therefore, hybrid scaffolds are often developed by combining both.^35^ PCL has generally been employed in combination with other materials. These scaffolds promoted cell growth and enhanced differentiation, forming aligned myotubes and allowing the exchange of nutrients and mechanical properties.^14,36,37^ For instance, Perez-Puyana et al. focused on the use of PCL in combination with elastin, collagen, and gelatin,^24,25^ while Kim et al.^38^ reported that scaffolds with nanofibers of PCL and gelatin can modulate myoblast differentiation better than scaffolds with only PCL nanofibers. Scaffolds with the combination of PCL with gelatin increased the myogenin expression, contributing to myoblast maturation and myotube formation.^25,39^

This research has focused on the use of PCL and gelatin scaffolds that were generated in the work of Perez-Puyana et al.^25^ The degree of alignment in the scaffolds was not an influential factor in cell viability (*P* = 0.267) and myoblast fusion (*P* = 0.116). Cell morphology revealed that aligned scaffolds produced significantly narrower myotubes than random scaffolds. This variable may also be influenced by the thickness of the scaffold nanofibers, with the size of the myotubes increasing as the size of the scaffold nanofibers increased, and the scaffolds with the greatest thickness being random scaffolds.^25^ Different studies^29,40^ have attempted to relate the size of electrospun scaffold fibers without finding definitive results. On the contrary, our small study could pave the way for the theory that fiber size might be related to the size of the formed myotubes. Wang et al.^41^ made scaffolds with nanofiber yarn. This work suggested that the diameter of the aligned yarn could enhance cell elongation. In this work, we could see something similar to these results, due to the higher nucleus aspect ratio in scaffolds with aligned orientation, whose diameter of fibers was thinner than random fiber scaffolds.

By studying myotube alignment, aligned scaffolds exhibited significantly greater fiber alignment (*P* < 0.001). Our research supports the theory described by previous work on the ordering of myotubes in the reorganization of nanofibers of the scaffolds.^13−16,29^ This is also supported by authors like Liao et al.,^29^ who demonstrated that aligned fibers can play a role in the C2C12 orientation. They proved that random fibers promoted nucleus elongation, while aligned fibers showed aligned actin filaments with an elongated cytoplasm. Additionally, Wang et al.^41^ suggested that aligned nanofibers could guide the cellular alignment and elongated myotube formation.

Regarding the functionality, we found that random scaffolds had a contraction to the stimulus that may suggest that these may be the best option for clinical applications, such as musculoskeletal regeneration. This may be related to what was visualized in the morphological study, where we could see that the random scaffolds had a maturation greater than that of the aligned scaffolds. However, other factors such as fiber alignment, which are not sufficiently high in this type of scaffold, should be taken into account.

In addition to this, we measured the luminescence increase at the first and second peaks to know the cell fatigue suffered and the increase of luminescence after the stimuli. It has also been seen that despite the contraction that all scaffolds can undergo, there is certainly greater fatigue in random scaffolds after the first stimulus. This fatigue may be greater due to the greater contraction in the first stimulus.

The differentiation of skeletal muscle cells is controlled by the myogenic regulatory factors, which are myogenic factor 5, myogenic differentiation antigen (MyoD), myogenin (MYOG), and myogenic factor 4 (MRF4). However, there are different mechanisms involved in the proliferation and differentiation of these cells, such as mTOR, Notch, and transforming growth factor (TGF-β).^42^ Mechanical stimulation has been demonstrated as another factor of activation of different processes involved in the differentiation of skeletal muscle cells. Nagai et al.^43^ demonstrated the phosphorylation of extracellular regulated kinase, which is a mitogen-activated proteinkinase,^44^ induced by mechanical stimulation. Aguilar-Agon et al.^45^ studied the mechano-regulation of different gene transcriptions and observed an increase in some of them, such as IGF-1 mRNA, phosphorylation of Akt, p70S6K, and 4EBP-1. Stretch patterns that resemble developmental stages in vivo have been shown to increase myogenic gene expression, fiber size, and multinucleation, enhancing myotube organization within scaffolds in the direction of stimulation and increasing contractile function.^3,46,47^

We did not demonstrate that mechanical stimulation had an effect on the viability of random (*P* = 0.398) and aligned scaffolds (*P* = 0.273). The current literature does not conclude what effect mechanical stimulation may have on this variable.^31^ Mechanical stimulation significantly increased the fiber diameter of both random scaffolds (*P* < 0.001) and aligned scaffolds (*P* < 0.001). This is because the stimulation signals by recreating the cellular environment of the tissue promote fiber maturation. This result supports Powell et al’s work.^32^ Furthermore, alignment was slightly increased in aligned scaffolds (*P* = 0.023), as detailed by Ahmed et al.^48^ and Okano et al.^49^ However, we were unable to demonstrate that mechanical stimulation rearranged myotubes in random scaffolds (*P* = 0.700).

Additionally, previous works have studied cell circularity and its relationship with cell substrate rigidity.^50,51^ In our case, we chose to focus on the effect of mechanical stimulation on cell circularity. To a lesser extent, mechanical stimulation also caused the circularity of nucleus in aligned scaffolds to be reduced (*P* < 0.001), whereas in random scaffolds, it increased (*P* < 0.001). On the contrary, the aspect ratio was reduced in the aligned scaffolds (*P* < 0.001), whereas it increased in the random scaffolds (*P* < 0.001). These results in aligned scaffolds can support the results presented by Ahmed et al.^52^ where they found that the nucleus of the cells was elongated in the same orientation of the mechanical stimulation. Despite this, mechanical stimulation significantly increased the nuclear density within the myotubes of both groups (*P* = 0.004 in random scaffolds; *P* = 0.004 in aligned scaffolds), i.e., the myotubes of these stimulated scaffolds presented a higher amount of cells that could increase the functionality of the created tissue. This is consistent with what was found in the literature, where some authors support the idea that static culture systems are not able to deliver nutrients to the cells. Therefore, external stimulation, such as mechanical or magnetic stimulation, can significantly increase cell proliferation and differentiation.^53^ All this evidence can highlight the positive effect that the use of mechanically stimulated PCL and gelatin scaffolds can have by increasing viability, thickness, nuclear fusion, and targeting.

Finally, the stimulated scaffolds were subjected to a functional test. In random scaffolds, we could see some contractions; however, these responses were not synchronized with the stimulus. In aligned scaffolds, after the first stimulus, the mechanically stimulated scaffolds show a high peak of luminescence. After the second stimulus, the luminescence is practically nonexistent compared to that of the control group. Thus, we found that the scaffolds with mechanically stimulated alignment had higher fatigue at the second stimulus than those without stimulated scaffolds. We consider that the higher fatigue presented by the mechanically stimulated scaffolds may be related to the recovery time of the cells from the previous stimulus, a third stimulus was given after 3 and a half min of rest, and the luminescence increased considerably. Visualizing the luminescence graph, the mechanically stimulated scaffolds have a higher and better-synchronized increase and are even higher than those of the control aligned scaffolds and mechanical random scaffolds. This indicates that the contraction was more pronounced in the mechanically stimulated scaffolds, which underwent a greater contraction in response to the stimuli. This is in line with what was found in the literature and what was demonstrated in our work, regarding how mechanical stimulation leads to a higher cell differentiation of myoblast, as well as to an upregulation of contractile proteins.^29^ Aligned mechanically stimulated scaffolds were electrostimulated to know their response to this new stimulus. We could see the good response of the scaffolds to the stimuli at low frequencies. However, at a frequency of 2 Hz, the response was not evident. This may be due to the depolarization time of the cell membranes, which may be slower than the rate at which the stimuli change and does not allow calcium to enter the cells. Previous works showed the contraction behavior of C2C12 with a biphasic pulse at 1 Hz, increasing the fluorescence intensity due to the transient motion of calcium.^54^ The cultures were found to remain functional after this, as they showed a response to 1 Hz and high K^+^ solutions. All of these findings on the functionality of mechanically stimulated aligned scaffolds could be related to increased viability, thickness, nuclear fusion, targeting, and actin expression.

Given the above considerations, our results support the hypothesis put forward by Liao et al.^29^ and Candiani et al.^30^ following their results on the accumulation of proteins typical of myotubes. Liao et al.^29^ demonstrated that stimulation could increase the upregulation of contractile proteins. Candiani et al.^30^ determined that behind this accumulation could be cell hyperplasia due to increased cell proliferation prior to differentiation processes and cell hypertrophy with increased incorporation of nucleus into myotubes or stretch-mediated gene regulation leading to increased cytoskeleton protein synthesis. Despite this, cell proliferation may be increased in the mechanically stimulated samples. Other possible mechanisms that could account for the accumulation of cytoskeletal proteins in mechanically stimulated scaffolds are the sustained release of mechanically induced growth factors, which positively influence protein synthesis and prevent myotube atrophy, and the enhanced diffusion between scaffold fibers associated with the stimulation, which allows catabolite/anabolite exchange. In particular, nutrients that have penetrated the scaffold can be pushed toward its center with mechanical perturbation, thus contributing to an overall improvement of cellular metabolic capacities.

Some of the limitations of this work include more assays in the protein expression of skeletal muscle such as myosin heavy chain, MyoD, or myogenin. In this work, we used C2C12 since this cell line is easy to work with. C2C12 are seeded as proliferative myoblast; when reaching confluence, myoblasts begin to differentiate, fusing into elongated, multinucleated ones,^55^ but occasionally, the use of C2C12 myoblasts might be limited by their low differentiation potential.^56^ Therefore, the study results could seem more robust if a different cell type, such as human mesenchymal cells, were used to pave the way for future steps, including clinical translation. The aligned scaffolds had a thinner nanofiber thickness compared to that of the other scaffolds, which might have led to thinner myotubes and could be seen as a limiting factor. Despite the alignment, perhaps a higher concentration of biomaterials could lead to an increase in nanofiber thickness and, consequently, a greater thickness of the myotubes, affecting their nuclear density and functionality. We could not determine what happened to the gelatin. It is well-known that gelatin loses mechanical properties at physiological temperatures due to solubility.^57^ Different works^58^ crosslink the gelatin scaffolds, but also, others do not crosslink them.^59,60^ However, Jang et al.^61^ suggested that the loss of the mechanical properties could happen even in the crosslinked gelatin scaffolds. This approach will be studied in future works. Another limitation is related to the contamination of cell cultures, which increased the difficulty of working with the bioreactor, requiring assembly to be carried out under a laminar flow hood.

In the future, combined stimulation should be studied to evaluate whether it would benefit skeletal muscle development, as previous studies have reported the possible influence of electromechanical stimulation on differentiation in 3D cultures.^29,30^ Since this is a tissue created ex vivo, in the future, in vivo behavior could be studied through a preclinical assay. In this way, it would be known whether the functional tissue created can repair the tissue to be repaired or, on the contrary, cell migration would be observed, as in previous work by the group.^24^ This would help us to determine whether the tissue created ex vivo would be useful or if the incorporation of a scaffold would be sufficient. It would also be a subject of study if the created tissue should be stimulated in some way after implantation in the model as previous studies described.^62^ Finally, concerning the culture time, it would be interesting to study the effect in shorter periods at 2 weeks of stimulation as Candiani et al. did.^30^

## Conclusions

Despite not being able to demonstrate the protein expression of skeletal muscle, we could use electrospun scaffolds stimulated with a bioreactor with mechanical stimulation to produce functional skeletal muscle tissue with the ability of contraction. The PCL scaffold together with gelatin fabricated by electrospinning was ideal, as well as their mechanical stimulation versus other stimuli. We found that these scaffolds meet ideal regeneration conditions, such as increased fiber thickness, alignment, nuclear fusion, nuclear differentiation, and functionality.

## Supporting Information Available

Response against electrostimulation of a mechanically stimulated scaffold (MP4)
